# Role of surface charge and oxidative stress in cytotoxicity of organic monolayer-coated silicon nanoparticles towards macrophage NR8383 cells

**DOI:** 10.1186/1743-8977-7-25

**Published:** 2010-09-11

**Authors:** Sourav Bhattacharjee, Laura HJ de Haan, Nynke M Evers, Xue Jiang, Antonius TM Marcelis, Han Zuilhof, Ivonne MCM Rietjens, Gerrit M Alink

**Affiliations:** 1 Division of Toxicology, Wageningen University, PO Box 8000, 6700 EA Wageningen, The Netherlands; 2 Laboratory of Organic Chemistry, Wageningen University, Dreijenplein 8, 6703 HB Wageningen, The Netherlands

## Abstract

**Background:**

Surface charge and oxidative stress are often hypothesized to be important factors in cytotoxicity of nanoparticles. However, the role of these factors is not well understood. Hence, the aim of this study was to systematically investigate the role of surface charge, oxidative stress and possible involvement of mitochondria in the production of intracellular reactive oxygen species (ROS) upon exposure of rat macrophage NR8383 cells to silicon nanoparticles. For this aim highly monodisperse (size 1.6 ± 0.2 nm) and well-characterized Si core nanoparticles (Si NP) were used with a surface charge that depends on the specific covalently bound organic monolayers: positively charged Si NP-NH_2_, neutral Si NP-N_3 _and negatively charged Si NP-COOH.

**Results:**

Positively charged Si NP-NH_2 _proved to be more cytotoxic in terms of reducing mitochondrial metabolic activity and effects on phagocytosis than neutral Si NP-N_3_, while negatively charged Si NP-COOH showed very little or no cytotoxicity. Si NP-NH_2 _produced the highest level of intracellular ROS, followed by Si NP-N_3 _and Si NP-COOH; the latter did not induce any intracellular ROS production. A similar trend in ROS production was observed in incubations with an isolated mitochondrial fraction from rat liver tissue in the presence of Si NP. Finally, vitamin E and vitamin C induced protection against the cytotoxicity of the Si NP-NH_2 _and Si NP-N_3_, corroborating the role of oxidative stress in the mechanism underlying the cytotoxicity of these Si NP.

**Conclusion:**

Surface charge of Si-core nanoparticles plays an important role in determining their cytotoxicity. Production of intracellular ROS, with probable involvement of mitochondria, is an important mechanism for this cytotoxicity.

## Introduction

Silicon (Si) is conventionally regarded as a non-toxic semiconductor material and Si NP are proposed as an alternative for the highly toxic heavy metal quantum dots in biological applications such as in food industry and bioimaging [1]. However, once exposed to an aerobic atmosphere, Si NP readily get oxidized to silica (silicon dioxide; SiO_2_) [2], which is reported to result in cytotoxicity [3]. The cytotoxicity of silica nanoparticles has been reported to be size dependent [4]. Data on the actual toxicity of silicon NP (Si NP) are, however, scarce.

Recently, we developed a method for the gram-scale synthesis of Si NP [5], which can be coated with a covalently bound organic monolayer with different surface charges [6-8]. These nanoparticles have a silicon core of 1.6 ± 0.2 nm as determined by TEM [7,8]. By attaching alkyl chains to the surface of the Si core with amine (NH_2_), azide (N_3_) and carboxylic acid (COOH) terminal moieties, Si NP with respectively positive (Si NP-NH_2_), neutral (Si NP-N_3_) and negative (Si NP-COOH) surface charges can be obtained. This coating prevents the oxidation of Si NP to SiO_2_. Also the influence of surface charges on the cytotoxicity remains largely unresolved, although there are several research articles pointing at a possible role of surface charge in cellular uptake and/or cytotoxicity of nanoparticles. Oskuee et al.[9], for example, reported a decrease in cytotoxicity with decreasing positive surface charge of polyethyleneimine NP-s. Sayin et al.[10] found that positively charged N-trimethyl chitosan NP-s were more cytotoxic than their negatively charged counterparts.

A brief overview of some recent articles pointing at the possible influence of surface charge of nanoparticles on their cellular uptake and/or cytotoxicity is given in Table 1, which summarizes the findings reported by different groups. The findings are based on different types of particles functionalized with different chemical groups. A consensus regarding the role of surface charge on cytotoxicity of nanoparticles is therefore hard to reach. Some research groups [11,12] observed cytotoxic effects of positively charged nanoparticles. Mayer et al.[13] reported activation of the complement system and increased hemolysis in blood samples collected from healthy donors after being exposed to positively charged polystyrene nanoparticles. Some recent publications [14-20] reported different effects of surface charges on cytotoxicity, including a higher cytotoxicity of cationic nanoparticles as compared to anionic nanoparticles. Gupta et al.[21] recently observed a reduced cytotoxicity for nanoparticles with a positive surface charge. On the other hand, other research groups [22,23] failed to observe any significant effect of surface charge of nanoparticles on their cytotoxicity.

**Table 1:** Brief overview of recent publications pointing at a possible role of surface charge in interaction of nanoparticles with cells

Citation (Year)	Nanoparticle tested	Size of nanoparticle (nm)	Cell Line tested (*in vitro/in vivo*)	Endpoints studied	Results/Inferences
Ruizendaal et al. (2009) [6]	Si NP with amine (+), azide (neutral) and acid (-) surface functionalization	1.6 ± 0.2	Caco-2	MTT, BrdU	Positively charged Si NP-NH_2 _more cytotoxic than neutral Si NP-N_3_. Negatively charged Si NP-COOH did not show toxicity.

Geys et al. (2009) [11]	Quantum dots (amine terminated, neutral, carboxylate terminated)	25	Primary alveolar epithelial cells	MTT, TEER, sodium fluorescein leakage, confocal microscopy	Surface charge did not show any influence on translocation through the cell line.

Corsi et al. (2009) [12]	Iron based magnetic nanoparticles	7 ± 3	MCF7 carcinoma cells	MTT	Anionic nanoparticles were spontaneously internalized. Cationic ones were taken up by clathrin receptor mediated endocytosis.

Sadiq et al. (2009) [13]	Aluminium oxide	179	*E. coli*	Bacterial growth, Infrared spectroscopy	Interaction between positively charged particles and bacteria was found

Xu et al. (2009) [14]	Hemoglobin loaded polymeric NPs	< 200	(MPM) cell line from SD mice	MTT, *in vivo *biodistribution and clearance of NPs	No influence of surface charge on cytotoxicity was observed.

Nafee et al. (2009) [15]	Chitosan modified PLGA	between 150 and 250	COS-1, A549, Calu-3	MTT, LDH, ATP, TEER, SFM	Higher zeta potential was connected with lower toxicity for COS-1, while no effect of surface charge was found for A549 cells.

Pathak et al. (2009) [16]	Branched polyethylenimine with chondroitin sulphate	between 80 and 190	HeLa, HepG2	MTT, DNA release, protein adsorption, confocal microscopy, gene transfection, radiolabelling, biodistribution, scintigraphy	Reduction in positive charge by increasing the percentage of chondroitin sulphate decreases cytotoxicity.

Mayer et al. (2009) [17]	Polystyrene	26, 34, 62, 160, and 220	Human blood	Flow cytometry for thrombocyte and granulocyte activation, plasma coagulation assay, light microscopy, membrane integrity assay, C3a and C5a ELISA, hemolysis	Positive surface charge led to complement activation.

Zhang et al. (2009) [18]	Amine, PEG and carboxylic acid terminated CdSe quantum dots with ZnS shell	12×6	HEK	TEM, quantification of quantum dot fluorescence, immunostaining	Uptake of amine-terminated quantum dots proceeds by caveolin/clathrin pathway, while that of carboxylic acid terminated ones proceed by GPCR pathway

Nam et al. (2009) [19]	Glycol chitosan with 5β cholanic acid	359	HeLa	Cellular uptake studies	Increase in positive charge results in enhanced uptake and distribution by clathrin, caveolin receptor mediated, macropinocytosis.

Gupta et al. (2009) [20]	Polyacrylic acid and YFa	83 ± 8	HepG2, N2a, HEK293	MTT, RBC, WBC, platelet count from blood samples, *in vitro *peptide release study	Positively charged particles do not have any toxic behaviour.

Kim et al. (2008) [21]	Quantum dot nanocomposites	104.5 ± 7.8	SNB19	Scanning electron microscopy, TIRF, cell viability	Cationic coating at basic pH, makes the NPs more biocompatible.

Hauck et al. (2008) [22]	Gold nanorods with polyelectrolyte surface coating	18×40	Vi-cell, HeLa	TEM, Trypan Blue exclusion, gene expression	Only CTAB (positively charged) coated particles were toxic in absence of FCS.

Orr et al. (2007) [23]	Silica	100, 500	C10 (alveolar type II epithelial cell line)	X-ray diffraction, TEM, DIC, SEM	Positively charged particles can reach the cells through filopodia and microvilli-like structures. Positive surface charge and intact actin filaments are essential for retrograde movement of the particles.

Previously, we reported the synthesis and cytotoxic effects of differently charged Si NP towards human colonic adenocarcinoma derived Caco-2 cells [6]. Positively charged Si NP-NH_2 _exerted the highest toxicity, whereas the negatively charged Si NP-COOH were hardly toxic and the neutral Si NP-N_3 _showed intermediate toxicity towards the Caco-2 cells. Unfortunately, investigations regarding the mechanisms of cytotoxicity of nanoparticles are quite limited. Also, different research groups use different experimental models. Nevertheless, oxidative stress is proposed as one of the most important mechanisms for NP mediated toxicity [24-33]. In these studies a wide range of nanoparticles was tested in different cellular models. Still, a concerted study on the effect of surface charge on the production of intracellular ROS and subsequent oxidative stress and cytotoxicity is not available. Therefore, in the present study we systematically investigated the role of NP surface charge and oxidative stress in the rat alveolar macrophage NR8383 cell line. This cell line provides an adequate model system for studying the effect of NP on phagocytic cells, which can clarify possible effects on the innate immune response. In the current study the mechanism underlying the differential cytotoxicity of the various Si NP is investigated, with a focus on the possible role for the formation of intracellular ROS and oxidative stress. To this end the production of intracellular ROS in both Caco-2 and NR8383 cells exposed to increasing concentrations of the differently charged Si NP was investigated. Also the degree of protection provided by pre-incubation of the cells with the antioxidants vitamin E and vitamin C and the possible role of Si NP-induced mitochondrial activity in the production of the intracellular ROS was studied. The results indicate how the surface charge of the NP influences their capability to induce intracellular ROS formation and oxidative stress and point at a role of mitochondria in this NP-induced ROS production at the subcellular level.

## Results

**Figure 1:**
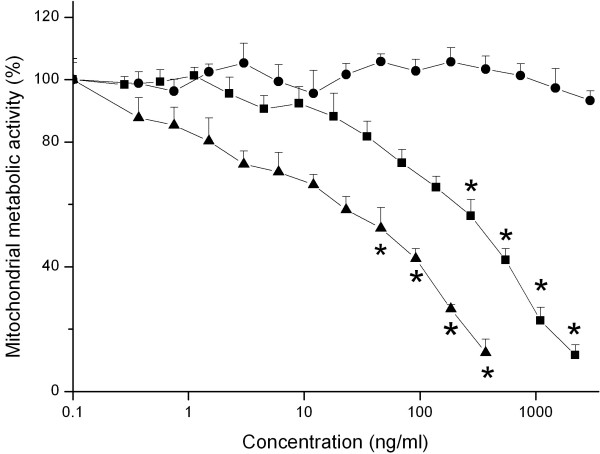
**Effect of 24 hours exposure of NR8383 cells to increasing concentrations of Si NP-NH_2 _(black triangle), Si NP-N_3 _(black square) and Si NP-COOH (black dot) on mitochondrial metabolic activity (measured by the MTT assay)**. Error bars show standard error of mean (n = 3). The asterisk (*) sign signifies *P *< 0.05.

Figure 1 shows the cytotoxic effect of increasing concentrations of various Si NP on NR8383 cells as detected by the reduction in the mitochondrial metabolic activity (MTT assay) of these cells after 24 hours exposure. Si NP-NH_2 _showed the highest cytotoxicity (EC50 value = 12 ng/ml) followed by Si NP-N_3 _which were cytotoxic at relatively higher concentrations (EC50 value = 270 ng/ml). Si NP-COOH failed to show any cytotoxicity up to concentrations of 3000 ng/ml (Table 2).

**Table 2:** The EC50 values of different Si NP obtained from experiments reported in this article

Assay	Parameter	Reference figure	Si NP-NH_2_	Si NP-N_3_
MTT	Mitochondrial metabolic activity	Figure 1	12	270

Phagocytosis Index	Induction Factor	Figure 2	60	320

DCFH-DA	Intracellular ROS production	Figure 3A (NR8383)	22	170
		Figure 3B (Caco-2)	18	310

DCFH-DA	ROS production (from isolated mitochondrial fraction)	Figure 4	80	1050

MTT (in presence of antioxidants vitamin E and vitamin C)	Mitochondrial metabolic activity	Figure 5 (for Vitamin E)	60	310
		Figure 5 (for Vitamin C)	32	510

**Figure 2:**
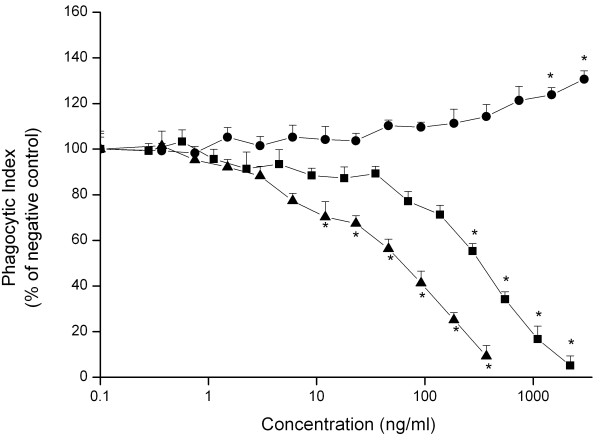
**Effect of 24 hours exposure of NR8383 cells to increasing concentrations of Si NP-NH_2 _(black triangle), Si NP-N_3 _(black square) and Si NP-COOH (black dot) on phagocytosis (measured as the phagocytic index)**. Error bars show standard error of mean (n = 3). The asterisk (*) sign signifies *P *< 0.05.

The effects of increasing concentrations of the differently charged Si NP on the phagocytic index of NR8383 cells upon 24 hours of exposure are shown in figure 2. Exposure to Si NP-NH_2 _and Si NP-N_3 _resulted in a concentration-dependent decrease in the phagocytic index. The EC50 value for Si NP-NH_2 _was 60 ng/ml and for Si NP-N_3 _it was 320 ng/ml (Table 2). Exposure to Si NP-COOH did not result in a decrease in phagocytosis index, but surprisingly, even resulted in a steady increase in the phagocytosis index up to 130 % of the control values at a concentration of 3000 ng/ml.

**Figure 3:**
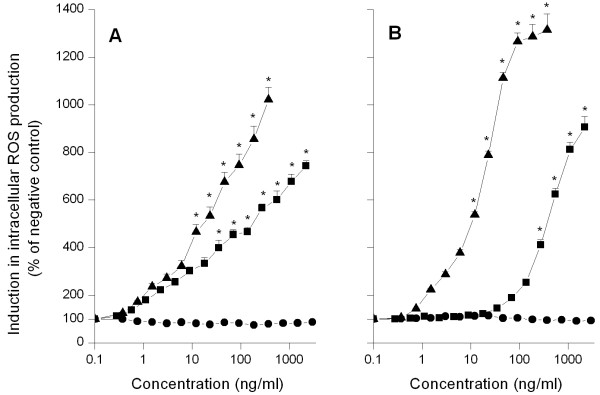
**Induction of intracellular ROS production (measured using the DCFH-DA assay) in NR8383 (A) and Caco-2 (B) cells after 24 hours exposure to increasing concentrations of Si NP-NH_2 _(black triangle), Si NP-N_3 _(black square) and Si NP-COOH (black dot)**. Error bars show standard error of mean (n = 3). The asterisk (*) sign signifies *P *< 0.05.

In figure 3A the intracellular ROS production (measured by DCFH-DA assay) in NR8383 cells after 24 hours exposure to various Si NP is shown. Exposure of NR8383 cells to Si NP-NH_2 _resulted in a dose-dependent increase in intracellular ROS production (EC50 value = 22 ng/ml). Upon exposure to Si NP-N_3 _also an increase in intracellular ROS production was observed (EC50 value = 190 ng/ml), although the rate of intracellular ROS production was lower than that observed upon exposure of the NR8383 cells to Si NP-NH_2 _(Table 2). Upon exposure of the NR8383 cells to Si NP-COOH no increase in intracellular ROS production was observed.

In figure 3B the intracellular ROS production (measured by DCFH-DA assay) in Caco-2 cells after 24 hours exposure to various Si NP is shown. Upon exposure to Si NP-NH_2 _intracellular ROS production started at low concentrations and then grew rapidly reaching a plateau at concentrations above 46 ng/ml (EC50 value = 18 ng/ml; Table 2). For Caco-2 cells exposed to Si NP-N_3_, intracellular ROS production increased significantly at concentrations above 50 ng/ml and increased up to higher concentrations (EC50 value = 310 ng/ml; Table 2). For Caco-2 cells exposed to Si NP-COOH, no increase in intracellular ROS production was observed.

**Figure 4:**
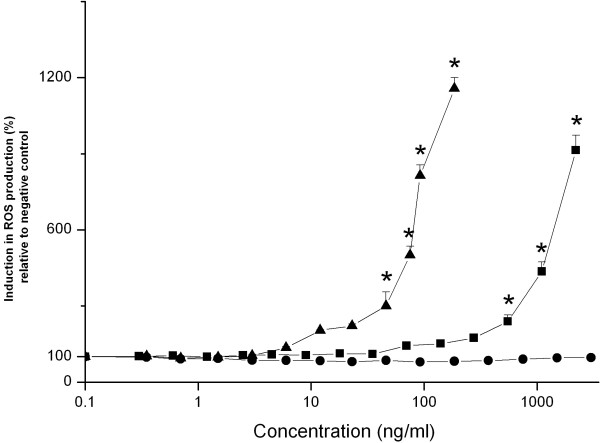
**Induction of ROS production (measured using the DCFH-DA assay) upon 90 minutes incubations of an isolated rat liver mitochondrial fraction with increasing concentrations of Si NP-NH_2 _(black triangle), Si NP-N_3 _(black square) and Si NP-COOH (black dot)**. Error bars show standard error of mean (n = 3). The asterisk (*) sign signifies *P *< 0.05.

In figure 4 ROS production (measured by DCFH-DA assay) upon 90 minutes incubation of an isolated rat liver mitochondrial fraction with different Si NP is shown. Incubation with Si NP-NH_2 _resulted in a concentration-dependent increase in ROS production, which started at concentrations as low as 6 ng/ml. The calculated EC50 value for Si NP-NH_2 _induced mitochondrial ROS production was 80 ng/ml (Table 2). Incubation of the mitochondrial fraction with Si NP-N_3 _resulted in a concentration-dependent increase in ROS formation starting at 90 ng/ml, with an EC50 value of 1050 ng/ml (Table 2). No increase in ROS production was found for the Si NP-COOH even for concentrations as high as 3000 ng/ml.

**Figure 5:**
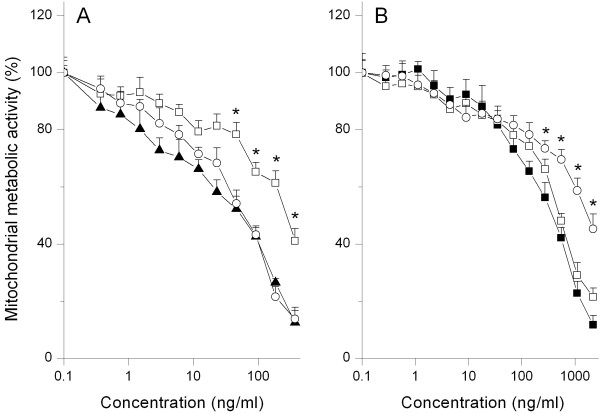
**Protective effect of preincubation of NR8383 cells with vitamin E (open square) and vitamin C (open circle) against cytotoxicity (measured using the MTT assay) detected after 24 hours exposure to serial dilutions of (A) Si NP-NH_2 _(black triangle) and (B) Si NP-N_3 _(black square)**. Error bars show standard error of mean (n = 3). The asterisk (*) sign signifies *P *< 0.05 (compared to data without antioxidants).

In figure 5A the effects are depicted of the antioxidants vitamin E and C on the cytotoxicity in NR8383 cells upon 24 hours exposure to Si NP-NH_2_. Preincubation of the NR8383 cells with vitamin E resulted in a significant reduction in the Si NP-NH_2_-induced cytotoxicity with EC50 values of 12 ng/ml and 60 ng/ml (*p *< 0.05) for NR8383 cells not preincubated with antioxidants (control) and cells preincubated with 100 μM vitamin E, respectively (Table 2). Although a higher EC50 value of 32 ng/ml was found for NR8383 cells preincubated with 1 mM of vitamin C in comparison to NR8383 cells not preincubated with antioxidants (control) the magnitude of reduction in cytotoxicity for 1 mM vitamin C was less than preincubation of NR8383 cells with 100 μM vitamin E (EC50 value = 60 ng/ml; Table 2).

The effects of vitamins E and C on the cytotoxic effects of Si NP-N_3 _in NR8383 cells are shown in figure 5B. For cells exposed to Si NP-N_3_, vitamin C offered a significant protection, whereas preincubation of the cells with vitamin E did not result in protection against the cytotoxicity of the NP. The corresponding EC50 values for Si NP-N_3 _were 270 ng/ml, 310 ng/ml and 510 ng/ml (*p *< 0.05), respectively, for NR8383 cells not preincubated with antioxidants (control), cells preincubated with 100 μM vitamin E, and cells preincubated with 1 mM vitamin C.

## Discussion

The surface charge of nanoparticles plays an important role in their interaction with cells and cytotoxicity. In this paper the role of surface charge on cytotoxicity is studied and mechanistic factors (like oxidative stress) causing this cytotoxicity are probed. The main conclusions are that positively charged Si NP-NH_2 _are more cytotoxic towards NR8383 cells both in terms of reducing mitochondrial metabolic activity and phagocytosis than neutral Si NP-N_3_, while negatively charged Si NP-COOH showed very little or no cytotoxicity. Si NP-NH_2 _produced most intracellular ROS followed by Si NP-N_3_, with no ROS produced by Si NP-COOH. Part of this ROS production probably originates from mitochondria, as a similar trend in ROS production was observed in NR8383 cells and in incubations of isolated rat liver mitochondrial fractions with Si NP. The Si NP-NH_2 _and Si NP-COOH are slightly more hydrophilic than Si NP-N_3_. However, since the difference in cytotoxicity and ROS production is much larger between Si NP-NH_2 _and Si NP-COOH than between Si NP-NH_2 _or Si NP-COOH and Si NP-N_3 _we conclude that surface charge is much more important than differences in hydrophilicity or hydrophobicity.

The role of ROS was also reflected in the protection against cytotoxicity by Si NP upon pretreatment of the NR8383 cells with vitamins E and C. Interestingly, pretreatment of the NR8383 cells with vitamin E showed more protection towards Si NP-NH_2 _than vitamin C, while vitamin C showed more protection towards Si NP-N_3_. The mechanism behind this difference may be related to the nature of the ROS formed and/or the location(s) and mechanism(s) of this ROS formation, although the results of the present study reveal that in both cases mitochondrial ROS production is likely involved. Pan et al.[34] previously reported different protective effects offered by different antioxidants (N-acetylcysteine, glutathione, triphenylphosphine monosulphonate and vitamin C) while studying the cytotoxic effects of gold nanoparticles on HeLa cells.

The relatively higher cytotoxicity of Si NP-NH_2 _as compared to Si NP-N_3 _or Si NP-COOH is in agreement with our previous study, where similar effects of these Si NP were observed on human colonic adenocarcinoma-derived Caco-2 cells [6]. Shiohara et al.[35] recently reported the synthesis and cytotoxicity of Si NP with different functionalized surfaces containing amine, epoxide, diene and diol terminated alkyl chains. These authors also reported a higher toxicity of amine-terminated Si NP as compared to the other Si NP. However, the functional groups of their Si NP were mostly different from our Si NP. Also, their Si NP were substantially larger (~4 nm in size), more polydisperse and prepared by a different synthetic technique. Goodman et al.[36] tested the cytotoxicity of cationic and anionic monolayer-coated gold nanoparticles on Cos-1 cells and found that cationic particles were toxic, whereas the anionic particles were not toxic at all, which matches our observation as well. Table 1 presents an overview of additional literature studies on nanoparticles, showing the role of surface charge on cellular interaction, including cellular uptake and cytotoxicity. It seems that the findings reported in these studies can be summarized to an overall match to our results since mostly positive charged nanoparticles are cytotoxic, although the cytological and experimental models used and endpoints studied in these reported studies are quite different from the ones we applied.

Our data also showed that positively charged Si NP-NH_2 _and the neutral Si NP-N_3 _decreased the phagocytic activity in NR8383 cells, but surprisingly reveal that the negatively charged Si NP-COOH increased the phagocytic index significantly. Although the precise mechanism of this phenomenon is not fully understood (see below), several indications have been reported in the literature that suggest that this is in fact not an unexpected phenomenon. One study reported comparable results for etoposide-incorporated tripalmitin nanoparticles with positive and negative surface charges [37]. That study showed that in rats, after 24 hours of intravenous administration, the concentration of positively charged particles in blood was 1.64 times that of negatively charged ones. Sengupta et al.[38] showed that etoposide-incorporated cationic liposomes attain a longer half-life in blood than anionic liposomes containing etoposide, due to a decreased phagocytic activity of macrophages for cationic particles that is not seen in the case of anionic particles. A similar charge-dependent increase (for negatively charged liposomes) or decrease (for positively charged liposomes) of phagocytosis of liposomes containing stearylamine was reported by Levchenko et al.[39]. Comparable results were reported by Aoki et al.[40] while investigating the disposition kinetics of liposomes with encapsulated methyl-2-amino-6-palmitoyl-D-glycoside. They found that the surface charge of liposomes is an important factor for phagocytosis of liposomes by the reticule-endothelial system: positively charged liposomes decreased phagocytosis, while negatively charged ones did not affect phagocytosis. It should be noted that all studies quoted above [37-40] were bioavailability studies and only potentially non-toxic nanoparticles were tested irrespective of their surface charge. Furthermore, in most of these studies no proper cytotoxicity tests were performed simultaneously with the bioavailability experiments.

As the Si NP-COOH proved to exert very little or no cytotoxicity at the tested concentrations, the induction of phagocytosis by negatively charged Si NP-COOH may be compared with similar findings reported in the above-mentioned studies. However, these findings should not be compared with our results on positively charged Si NP-NH_2 _which are cytotoxic within the tested concentration range. Hence, the decrease in phagocytic capability of NR8383 cells after exposure to positively charged Si NP-NH_2 _is the result of the positive surface charge and NP cytotoxicity but does not reflect an absence or induction in cellular uptake. A possible explanation for the induction of phagocytosis by negatively charged nanoparticles is given by Hernandez-Caselles et al.[41], who reported better binding of opsonin serum proteins with negatively charged nanoparticles than with positively charged nanoparticles. As a result, negatively charged nanoparticles get more covered with opsonin proteins, which stimulates phagocytosis by macrophages. It should be noted that the cell culture medium F12-K used in our experiments had fetal calf serum, which contained opsonin proteins (like immunoglobulins) in it as well.

In order to get more insight in the mechanism behind the cytotoxicity of Si NP towards NR8383 and Caco-2 cells we assessed the production of intracellular ROS. From our results it is evident that positively charged Si NP-NH_2 _were more efficient in producing intracellular ROS than the neutral Si NP-N_3_, which produced ROS only at much higher concentrations, and than negatively charged Si NP-COOH that did not produce any intracellular ROS at all. Xia et al.[42] did an extensive study on murine macrophage RAW 264.7 cells with a broad range of nanoparticles (including metal oxides like titanium dioxide, fullerols, carbon black and amine terminated polystyrene nanoparticles/PS-NH_2_) and found that only carbon black and cationic PS-NH_2 _were able to generate intracellular ROS production. ROS production by cationic PS-NH_2 _is in line with our study where Si NP-NH_2 _were also able to induce production of intracellular ROS. Stone et al.[43] also mentioned oxidative stress as an important mechanism for cytological injury caused by nanoparticles. Previously, Foucaud et al.[44] showed that carbon black ultrafine particles are capable of producing ROS in biologically relevant medium (like cell culture medium). Additionally, there are several other reports in which oxidative stress has been identified as an important mechanism for cytotoxicity of nanoparticles [45-47].

Subsequently, we investigated the capability of mitochondria to act as a source for nanoparticle-induced ROS production, since mitochondria have previously been recognized as a probable subcellular target of nanoparticles [48]. The results of our study reveal that mitochondria might indeed interact and contribute to the intracellular ROS production induced by NP. Karataş et al.[49] also reported that the mitochondrial membrane may be a target for NP interaction. They used an isolated mitochondrial fraction from A549 lung cancer cells incubated with negatively charged 13 nm gold nanoparticles, and studied the interaction with surface-enhanced Raman scattering (SERS). Using AFM and TEM they also demonstrated interaction of the gold nanoparticles with the mitochondrial membrane. There is one more report [34] in which 1.4 nm and 15 nm gold nanoparticles were tested on HeLa cells. The 1.4 nm gold nanoparticles were coated with triphenylphosphine sulphonate groups and their cytotoxic effect on the mitochondrial membrane potential (ΔΨ) was compared with bigger 15 nm gold nanoparticles. However, that study focused more on the size effect of gold nanoparticles on the mitochondrial membrane potential, and did not report the effect of surface charge of nanoparticles in interaction of the NP with the mitochondrial membrane. To the best of our knowledge, the present study is the first one to show production of ROS directly from mitochondria when exposed to NP. The observation that incubation of the mitochondrial fraction with positively charged Si NP-NH_2 _produced more ROS than incubation with neutral Si NP-N_3 _and negatively charged Si NP-COOH is in agreement with findings reported by Xia et al.[42]. With electron microscopy they saw that positively charged NH_2_-PS nanospheres completely disrupted the morphology of mitochondria after 16 hours of exposure, while negatively charged COOH-PS nanospheres did not cause any harm to the mitochondria. They also reported a dissipation in mitochondrial membrane potential (ΔΨ) with NH_2_-PS but not for COOH-PS nanospheres. These data suggest that the interaction of cationic NP with mitochondrial membranes exerts significant toxic effects.

## Conclusion

The present study demonstrates the importance of surface charge in the cytotoxicity of nanoparticles. Dose-dependent and charge-dependent production of intracellular ROS further emphasizes the role of oxidative stress as an important mechanism for cytotoxicity. Mitochondria are likely target organelles for nanoparticles in the production of intracellular ROS. The negative Si NP-COOH show potential for further biological applications, whilst positive Si NP-NH_2 _should be applied with care.

## Methods

### Silicon nanoparticles

Si NP-NH_2 _and Si NP-N_3 _were prepared and characterized by methods described by Rosso-Vasic et al.[8] and Si NP-COOH were prepared by the method described by Ruizendaal et al.[6]. All three NP were of comparable sizes with a mean core diameter of 1.6 ± 0.2 nm as determined by TEM [7,8] and all three Si NP are modified by about 25 surface groups [5]. Both Si NP-NH_2 _and Si NP-COOH were dissolved in ultrapure sterile water, while the stock solution of Si NP-N_3 _was dissolved in pure DMSO. The required final concentration ranges of all three Si NP were prepared by serial dilution with medium and amounted to 0.1 - 370 ng/ml for the Si NP-NH_2_; 0.1 - 2200 ng/ml for the Si NP-N_3_, and 0.1 - 3000 ng/ml for the Si NP-COOH which were the final tested concentration ranges for each of the assays described in this paper unless otherwise mentioned. No aggregation of these particles was observed with time in medium with DLS for at least 2 weeks and the solutions were vortexed at least for 1 minute before use. The solutions were filtered through 0.2 μm cellulose filters before cytotoxicity assays to maintain sterility. The amount of DMSO in the wells containing Si NP-N_3 _was calculated to be < 1% (v/v), at which concentration no cytotoxic effect of the DMSO was observed.

### Cell Lines and tissue fractions

#### NR8383 cells

Rat alveolar macrophage cells were obtained from ATCC (Manassas, VA). The cells were cultured in 150 cm^2 ^cell culture flasks with 25 ml F12-K culture medium (Gibco 21127) supplemented with 10% (v/v) heat-inactivated FCS in a humidified atmosphere containing 5% CO_2 _at 37°C.

#### Caco-2 cells

The human colonic adenocarcinoma cells were obtained from ATCC (Manassas, VA). The cell line was cultured in a humidified atmosphere containing 5% CO_2 _at 37°C in DMEM medium supplemented with 10% (v/v) heat-inactivated FCS, 1% (v/v) NEAA and 0.1% (v/v) gentamicin.

#### Isolation of the mitochondrial fraction from rat liver tissue

A Wistar rat of body weight 200 - 250 g was sacrificed by decapitation following anesthesia with isoflurane. The liver was excised at 4°C and homogenized in ice-cold saline using a Potter homogenizer with 20 - 25 strokes. The homogenized tissue was then centrifuged at 500 *g *for 10 minutes at 4°C. The supernatant was then removed and the pellet after being weighed was resuspended in ice-cold saline and centrifuged at 10.000 *g *for 10 minutes at 4°C. The supernatant was removed again and the pellet containing the isolated mitochondrial fraction was suspended in phosphate buffered saline (PBS) at 3 mg pellet/ml concentration with the addition of 0.4 mM glutamate and 0.4 mM NAD^+ ^as substrates for mitochondrial oxidative phosphorylation. The mitochondrial fraction was then kept in an incubator at 37°C with 5% CO_2 _atmosphere for 4 hours to ensure start of mitochondrial respiration.

### MTT assay

#### Cytotoxicity of Si NP

An NR8383 cell suspension was collected and centrifuged at 140 *g *for 5 minutes before resuspending the cell pellet in medium followed by counting and adjusting the cellular concentration to 2 × 10^5 ^cells/ml. The cells were then seeded in a 96-well plate (50 μl/well) and the plate was kept in a 5% CO_2 _incubator at 37°C for 24 hours. Next day 50 μl of serial dilutions of Si NP were added to the cells to obtain the required final concentrations of Si NP and then incubated for 24 hours. After 24 hours 5 μl of MTT solution in PBS (5 mg/ml) was added to each well and the plate was incubated for another 4 hours. Then 100 μl of pure DMSO was added to each well to dissolve the formazan crystals. Now the absorption of each well was measured at 562 nm in a 96-well plate reader and the background absorption at 612 nm [50] was subtracted. Mitochondrial metabolic activity for each concentration of Si NP was expressed as % of corresponding negative control reading. Medium without Si NP and medium with Triton-X (0.1%) were used as negative and positive controls respectively. Control experiments were done to exclude a possible reaction between MTT salt and Si NP. Additional control experiments were performed with stoichiometrically equivalent amounts of the coating materials (allylamine in case of Si NP-NH_2_, 10-undecenyl azide in case of Si NP-N_3 _and 1-butenoic acid in case of Si NP-COOH) to exclude cytotoxicity arising from the coating material molecules possibly remaining in the stock solutions of Si NPs as impurities. However, at these concentrations no cytotoxic effects were found for these compounds.

#### Protection by cellular preincubation with vitamin E

NR8383 cells were plated as described above (1 × 10^4 ^cells/well; 50 μl/well) in F12-K medium containing 100 μM vitamin E. After 24 hours 50 μl of serial dilutions of Si NP were added to the wells to obtain the required final concentrations of Si NP (the final concentration of vitamin E upon addition of the Si NP was reduced to 50 μM/well). Upon incubation for another 24 hours MTT reagent was added and the MTT assay was performed as described above. Control experiments were run with vitamin E only or Si NP only; both showed no activity in the MTT test.

#### Protection by cellular preincubation with vitamin C

NR8383 cells were plated in a 96-well plate (1 × 10^4 ^cells/well; 50 μl/well) in F12-K medium and after 22 hours vitamin C was added to reach a concentration of 1 mM vitamin C. After 2 more hours of incubation 50 μl of serial dilutions of Si NP were added to the wells to obtain the required final concentrations of Si NP (this reduced the final concentration of vitamin C to 500 μM). Upon incubation for another 24 hours MTT reagent was added and the MTT assay was performed as described above. Control experiments were run with vitamin C only or Si NP only; both showed no activity in the MTT test.

### Phagocytic Index measurement

A NR8383 cell suspension (8 × 10^6 ^cells/ml) was plated in a 96-well plate (50 μl/well) in F12-K medium, followed by addition of 50 μl/well of serial dilutions of Si NP to obtain the required final concentrations of Si NP. Plain F12-K medium without Si NP and medium containing 100 μM CuSO_4 _were used as negative and positive control respectively. After 24 hours the cells were exposed to yellow green fluorescent latex beads (1 μm size) at a ratio of beads to cells in each well of 50:1. After 4 hours of incubation counting samples were taken from the wells and viewed first under a fluorescent microscope to visualize the fluorescent beads, followed by bright field view to visualize the cells [see additional file 1]. Also samples were taken out of each well to assess the cell viability by Trypan Blue exclusion test. The phagocytic index was determined by calculating the average number of fluorescent beads phagocytosed per viable cell and expressed as % of the negative control. Medium without Si NP served as negative control.

### DCFH-DA assay

#### NR8383 cells

The cell suspension was adjusted to 2 × 10^5 ^cells/ml and seeded in a 96-well plate (50 μl/well) in F12-K medium. 50 μl/well of serial dilutions of Si NP in F12-K medium were added to obtain the required final concentrations of Si NP. A final concentration of 10 mM H_2_O_2 _was used as positive control and F12-K medium without nanoparticles as negative control. After 6 hours of exposure to the Si NP, 5 μl of a 20 mM solution of DCFH-DA were added to each well and the plates were incubated for another 18 hours in a 5% CO_2 _atmosphere at 37°C. The fluorescence was then measured on a fluorometer at 485 nm excitation and 538 nm emission wavelengths. The fluorescence induction factor for each concentration of Si NP was calculated by dividing the reading of each well by the average reading of the negative control and expressed as %. Control experiments were performed by incubating the Si NP at their test concentrations with DCFH-DA in the absence of cells to check the possibility of a positive fluorescence reading caused by reaction with Si NP alone.

#### Caco-2 cells

The cells were suspended in DMEM medium to a concentration of 1 × 10^5 ^cells/ml after trypisinization and were plated in a 96-well plate (100 μl/well). After 24 hours the cells were exposed to 100 μl/well of final concentrations of Si NP. Following another 6 hours of Si NP exposure, 5 μl of a 20 mM solution of DCFH-DA were added. The plate was further incubated for 18 hours before measurement of the fluorescence was carried out as described above. Control experiments were performed by incubating the Si NP at their test concentrations with DCFH-DA in the absence of cells to check the possibility of a positive fluorescence reading caused by reaction with Si NP alone.

#### Effect on isolated mitochondrial fraction

The isolated mitochondrial fraction (3 mg pellet/ml in PBS) was plated in a 96-well plate (50 μl/well) and serial dilutions of Si NP and 5 μl of DCFH-DA probe were added. The plate was incubated for 90 minutes at 37°C in a humidified 5% CO_2 _atmosphere. The plate was then measured at 485 nm excitation and 538 nm emission wavelength. Medium without Si NP and with 75 μM DNP in DMSO were used as negative and positive controls respectively. Results were expressed as % of negative control.

### Statistical analysis

Data were analyzed with Origin Pro (version 8.0) graphing software. For statistical analysis a student's *t*-test was performed and data with *P < 0.05 *(compared to negative control, except for Figure 5) are marked with an asterisk (*) sign. Each data point represents the average from three independent experiments (n = 3) and is presented as the arithmetic mean ± SEM.

## List of abbreviations

AFM: atomic force microscopy; ATCC: American type culture collection; ATP: adenosine triphosphate; BrdU: 5-bromo-2-deoxyuridine; C3a, C5a: complement component 3 and 5; CdSe: cadmium selenide; CO_2_: carbon dioxide; CuSO_4_: copper sulphate; DCFH-DA:2',7'-dichlorofluorescein-diacetate; DIC: disseminated intravascular coagulation; DLS: dynamic light scattering; DMEM: Dulbecco's modified eagle medium; DMSO: dimethyl sulfoxide; DNA: deoxyribonucleic acid; DNP: 2,4-dinitrophenol; EC50: half maximal effective concentration; ELISA: enzyme-linked immunosorbant assay; ETC: electron transport chain; FCS: fetal calf serum; F12-K: Ham's F12- medium with Kaighn's modification; FTIR: Fourier transform infrared spectroscopy; GPCR: G-protein coupled receptor; H_2_O_2_: hydrogen peroxide; LDH: lactate dehydrogenase; MTT: 3-(4,5-dimethylthiazol-2-yl)-2,5-diphenyltetrazolium bromide; ml: milliliter; mM: millimolar; μl: microliter; μM: micromolar; NAD: nicotinamide dinucleotide; NEAA: non-essential amino acids; ng: nanogram; NMR: nuclear magnetic resonance; PBS: phosphate buffered saline; PEG: polyethylene glycol; PLGA: poly (D,L-lactide-*co*-glycolide); RBC: red blood corpuscles; ROS: reactive oxygen species; RPM: rotations per minute; SEM: standard error of mean; SFM: scanning force microscopy; Si: silicon; Si NP: silicon nanoparticles; Si NP-COOH: carboxylic acid terminated silicon nanoparticles; Si NP-NH_2_: amine terminated silicon nanoparticles; Si NP-N_3_: azide terminated silicon nanoparticles; TEER: trans epithelial electric resistance; TEM: transmission electron microscopy; TIRF: total internal reflection fluorescence (microscopy); WBC: white blood corpuscles;

## Competing interests

The authors declare that they have no competing interests.

## Authors' contributions

SB, LHJDH, GMA, IMCMR conceived and designed the experiments. SB, NME (supervised by LHJDH) and XJ (supervised by SB) performed the experiments. ATMM and HZ supervised the synthesis of Si NP. SB analyzed the data and wrote the manuscript. ATMM, HZ, GMA and IMCMR supervised the project and corrected the manuscript. All authors read, corrected and approved the final manuscript.

## Supplementary Material

Additional file 1 **Fluorescence microscopy picture of NR8383 cells with phagocytosed 1 μm latex beads**. Click here for file
