# Supplementary material for: Role of surface charge and oxidative stress in cytotoxicity of organic monolayer-coated silicon nanoparticles towards macrophage NR8383 cells
Source: Part Fibre Toxicol. 2010 Sep 11;7:25. doi: 10.1186/1743-8977-7-25 (PMC2946263; doi:10.1186/1743-8977-7-25)
Supplement: Additional file 1 — Fluorescence microscopy picture of NR8383 cells with phagocytosed 1 μm latex beads. [file 1743-8977-7-25-S1.DOC]

**Additional file**

Fluorescence microscopy picture of NR8383 cells with phagocytosed 1 µm latex beads.


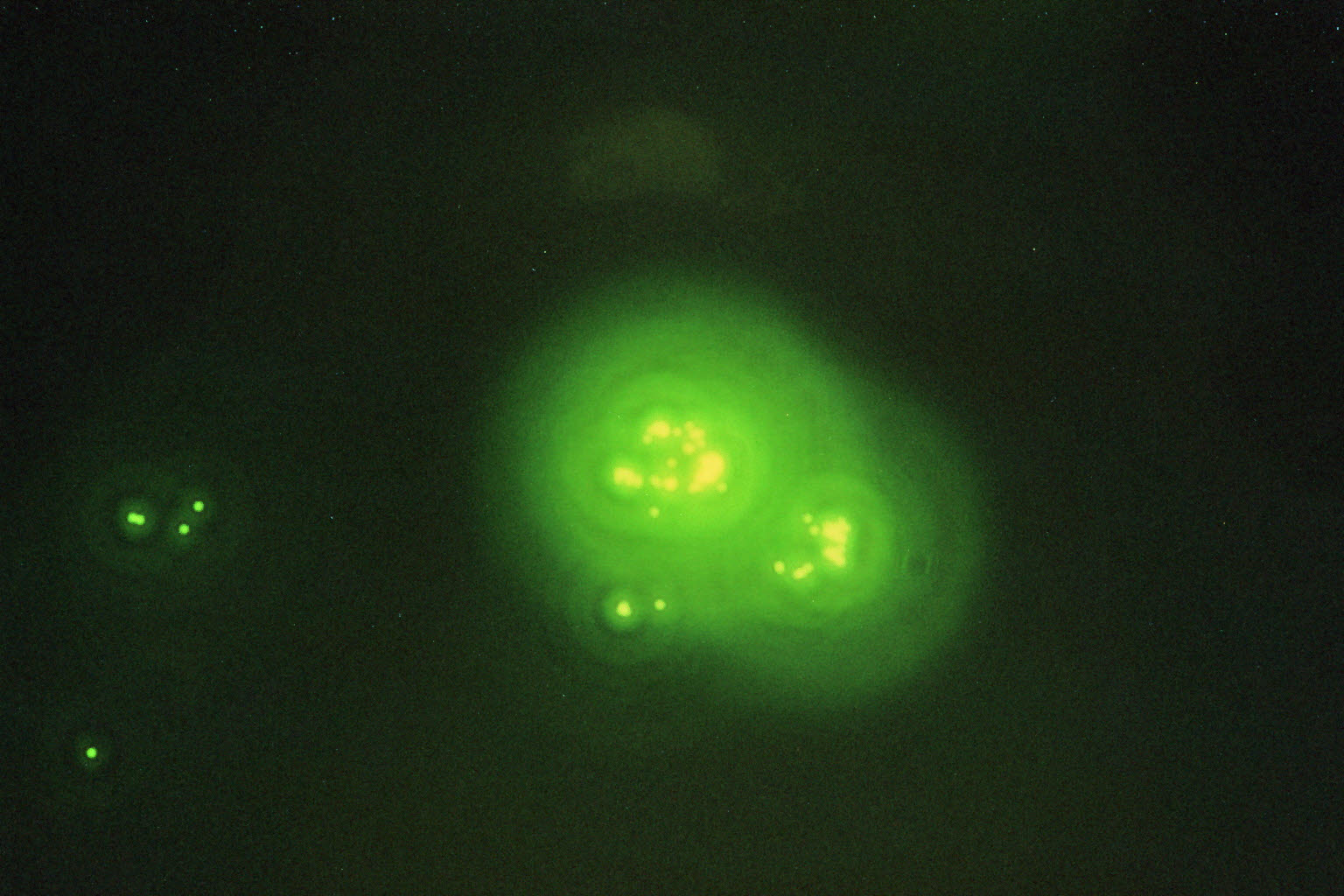


Belonging to paper:

**Role of surface charge and oxidative stress in cytotoxicity of organic monolayer-coated silicon nanoparticles towards macrophage NR8383 cells**

Sourav Bhattacharjee, Laura H. J. de Haan, Nynke M. Evers, Xue Jiang,

Antonius T. M. Marcelis, Han Zuilhof, Ivonne M. C. M. Rietjens, Gerrit M. Alink
